# Glucose Incorporated Graphite Matrix for Electroanalysis of Trimethoprim

**DOI:** 10.3390/bios12100909

**Published:** 2022-10-21

**Authors:** Rakesh R. Sawkar, Mahesh M. Shanbhag, Suresh M. Tuwar, Ravindra S. Veerapur, Nagaraj P. Shetti

**Affiliations:** 1 Department of Chemistry, Karnatak Science College, Dharwad 580001, India; 2 Department of Chemistry, K.L.E. Institute of Technology, Hubballi 580027, India; 3 Department of Metallurgy & Materials Engineering, Malawi Institute of Technology, Malawi University of Science and Technology, Limbe 5196, Malawi; 4 Department of Chemistry, School of Advanced Sciences, KLE Technological University, Vidyanagar, Hubballi 580031, India; 5 University Center for Research & Development (UCRD), Chandigarh University, Mohali 140413, India

**Keywords:** trimethoprim, glucose-carbon paste electrode, calibration curve, detection limit, excipients

## Abstract

The antibiotic drug trimethoprim (TMP) is used to treat bacterial infections in humans and animals, and frequently TMP is used along with sulfonamides. However, a large portion of TMP is excreted in its active state, which poses a severe problem to humans and the environment. A sensitive, rapid, cost-effective analytical tool is required to monitor the TMP concentration in biological and environmental samples. Hence, this study proposed an analytical methodology to analyze TMP in clinical, biological and environmental samples. The investigations were carried out using a glucose-modified carbon paste electrode (G-CPE) employing voltammetric techniques. Electrochemical behavior was examined with 0.5 mM TMP solution at optimum pH 3.4 (Phosphate Buffer Solution, I = 0.2 M). The influence of scan rate on the electro-oxidation of TMP was studied within the range of 0.05 to 0.55 V/s. The effect of pH and scan rate variations revealed proton transfer during oxidation. Moreover, diffusion phenomena governed the irreversibility of the electrode reaction. A probable and suitable electrode interaction and reaction mechanism was proposed for the electrochemical oxidation of TMP. Further, the TMP was quantitatively estimated with the differential pulse voltammetry (DPV) technique in the concentration range from 9.0 × 10^−7^ to 1.0 × 10^−4^ M. The tablet, spiked water and urine analysis demonstrated that the selected method and developed electrode were rapid, simple, sensitive, and cost-effective.

## 1. Introduction

Trimethoprim (TMP) is a prominent antibacterial drug consumed with a variety of sulfonamides, such as sulfamethoxazole, to treat various infections caused by bacteria [1,2]. TMP has been utilized to treat bacterial infections in humans and livestock. It efficiently inhibits dihydrofolate reductase, which is required for the formation of four hydrogen folic acids [3]. According to the World Health Organization (WHO), TMP is the basic drug that can be used effectively to treat and prevent respiratory, urinary and intestinal infections caused by bacteria [4]. TMP is frequently used in conjunction with sulphonamides to improve therapy efficacy. It is also available as a single drug, excluding sulfonamides. Antimicrobial resistance prompted the formation of TMP derivatives, which led to some health hazards to humans [5]. The use of TMP is not recommended in the first trimester during pregnancy and for people who have specific blood abnormalities. It can also lower creatinine clearance in renal tubules and result in dangerously low thrombocyte levels (cells that assist blood clots) by reducing folic acid levels and accompanying bone marrow blood cell development [6]. As per literature, a large portion of the TMP dose (50–70%) is excreted in its pharmacologically active state, causing toxicity in the aquatic habitat, particularly on the green algae, P. Subcapitata, and Daphnids [7]. As a result, there is a need for a new analytical method for monitoring the substance’s residues in the environment so that the hazardous effects of the drug’s residues can be efficiently assessed. However, environmental cleanup procedures or assistance with the medical diagnosis can both be used to undertake close monitoring [8,9].

The literature revealed that many approaches had been conducted and tested for the detection of TMP, and the most often stated determination method for TMP is liquid chromatography [10]. Apart from that, spectrophotometry [11], capillary electrophoresis [12], High performance liquid chromatography (HPLC) [13], potentiometry [14] and voltammetric methods are also reported [15,16,17,18]. However, most of these approaches are costly, need many solvents, and lack selectivity and sensitivity. A simple, cost-effective, and time-saving approach for TMP determination is required.

Electrochemical sensors have recently aroused the scientific community’s interest and have been frequently employed as sensitive detection techniques [19,20]. Rapid reaction, high sensitivity and selectivity, beneficial portability, simple sample preparation process, and cost-effective and simple design are the advantages of these approaches [21]. These characteristics are advantageous not only for detecting the bioactive material, but also for gaining insight into its metabolic processes and gaining a better knowledge of their interactions [22,23]. Electrochemical sensors are widely used practical appliances for detecting targeted species in biological samples, viz., blood, urine samples, etc. [24]. These techniques are particularly adapted in constructing miniaturized tools with a wide array of domestic and therapeutical applications [25]. Electrochemical sensors also provide the potential qualities needed for drug therapy diagnosis and monitoring. As a result, with further study and investment, these strategies may provide faster results for patients and physicians.

Glucose is commonly termed a monosaccharide observed predominantly in an environment with an appealing non-enzymatic oxidation mechanism. Glucose is oxidized to form gluconate and adsorbed on the electrode surface. The intermediate RCOO^−^ can interact with the analyte molecule, which elevates the aggregation of the molecule at the electrode surface [26]. It has a strong affinity for oxidizing organic compounds and has a higher adsorption potential on carbon powder [27]. Adsorption can improve the electrode’s sensitivity by intensifying the interaction between the analyte and the sensor’s surface. The most attractive properties of glucose, such as great mechanical strength, less toxicity, biocompatibility, and chemical inertness, have encouraged the utilization of glucose as a modifier material to enhance the sensitivity and the detection limit of the developing electrode.

On the other hand, graphite, which is insoluble in water, is a readily available material with the qualities required for the fabrication of electrodes, making it an economical, practical choice. Graphite has a very high melting point and is a good conductor of electricity, even in high-temperature processes, without changing state. Using glucose and graphite with a binder oil, i.e., paraffin oil, improves sensitivity in the detection of TMP.

In this research, a very efficient approach for the detection of TMP was proposed by fabricating a carbon paste electrode with glucose. As per the literature, TMP determination has not been carried out using G-CPE so far. The established sensor could be appropriate for identifying TMP in clinical and biological samples.

## 2. Materials and Methods

### 2.1. Materials and Solutions

TMP and glucose were acquired from Sigma Aldrich (Bengaluru, India). Throughout the study, double distilled water was utilized. Ortho-phosphoric acid (H_3_PO_4_), potassium dihydrogen phosphate (KH_2_PO_4_), trisodium phosphate (Na_3_PO_4_), and disodium phosphate (Na_2_HPO_4_) were purchased from HiMedia chemicals (Bangalore, India); they were used without being purified further for the preparation of phosphate buffer solution (PBS) of 0.2 M to obtain different pH values.

### 2.2. Instruments Used

The electrochemical analyzer CHI-1112C (USA) was employed to record the voltammograms with the assistance of a three-electrode compartment comprising an auxiliary electrode (platinum wire), reference (Ag/AgCl), and the working electrodes carbon paste electrode (CPE) and glucose modified carbon paste electrode (G-CPE). All the investigations were conducted in an electrochemical cell under controlled conditions at ambient temperature. The electrolyte solution’s pH was evaluated using a pH meter (Equiptronix model, Mumbai, India). The developed electrode matrix was characterized using an atomic force microscope (AFM-Nanosurf, Liestal, Switzerland), and a scanning electron microscope with energy dispersive X-ray spectroscopy (SEM EDS-JSM IT500, JEOL, Tokyo, Japan).

### 2.3. Fabrication of A Sensor

The carbon paste was developed by mixing powder graphite and mineral oil in 7:3 ratio (*w/w*%) using a mortar, and then homogenizing [28]. The resultant paste was filled in a Teflon pipe having a copper wire for electrical conductance, and the surface of the fabricated sensor was smoothened, followed by washing with double distilled water. The G-CPE was developed by adding glucose (0.05 g), along with graphite powder and mineral oil of the quantity mentioned above, and was homogenized. Later, it was used to fill a Teflon tube and washed. After each analysis, the paste was correctly eliminated, and fresh paste was used for individual measurements. The pre-treatment of a sensor was done to reduce background current by employing the cyclic voltametric (CV) technique within the potential of 0.4–1.4 V for 20 cycles in pH 3.4 of PBS.

### 2.4. Pharmaceutical and Urine Sample Preparations

Tablets (Bacstol 200, Bangalore, India) with TMP as the main content were procured from a nearby pharmacy and were crushed to obtain a powder form. A standard stock solution was arranged by mixing an appropriate mass of tablet powder in double-distilled water. Then, it was sonicated to achieve proper dissolution. A known volume of the prepared supernatant liquid with a varied concentration in electrolytic solution was taken for investigations. Clinical sampling and urine sample analysis were performed to verify the developed electrode’s sensitivity. Primarily the samples of urine were acquired from healthy individuals. The samples were then centrifuged at ambient temperature, and the supernatant solution was spiked with a known concentration of TMP. The differential pulse technique (DPV) technique was adopted for recovery studies. The content of TMP in pharmaceutical and biological samples was examined to determine the recovery at the proposed sensor.

### 2.5. Water Sample Analysis

The water sample was collected from Kelgeri lake (Dharwad, India), and the water sample was filtered to remove suspended pollutants. Then, the filtered sample was mixed with PBS (pH 3.4) solution in a 1:1 ratio, followed by spiking of a known amount of TMP concentrations for recovery studies.

### 2.6. Electroactive Area of G-CPE

In voltammetric analysis, the electrode surface plays a prominent role as the electrochemical oxidation/reduction occurs at the electrode vicinity. Hence, the surface area was estimated employing the Randle-Sevcik Equation (1), and voltammetric measurements were registered at various scan rates for 1.0 mM K_3_ [Fe(CN)_6_] in a standard test solution in 0.1 M KCl as a supporting electrolyte [29].

I_p_ = (2.69 × 10^5^) n^3/2^D_°_^1/2^ C*A υ^1/2^ (1)

where, I_p_ is peak current, n is number of electrons transferred, D_°_ is diffusion coefficient, C* is concentration, A is area of electrode and υ is scan rate. The active area of the fabricated electrode material was estimated to be 0.042 cm^2^ for nascent CPE and 0.074 cm^2^ for G-CPE, respectively.

## 3. Results and Discussions

### 3.1. Characterization of the Modifier

**Figure 1:**
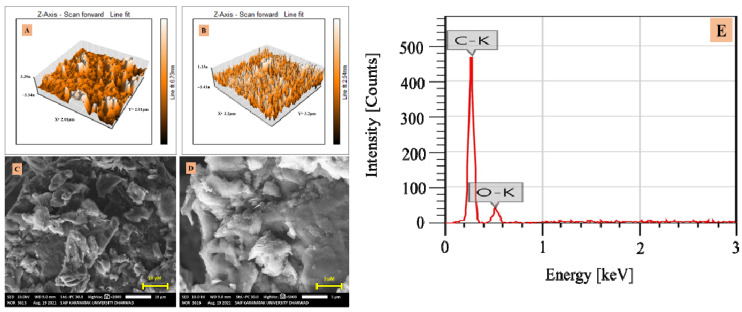
AFM image of (**A**) CPE and (**B**) G-CPE, (**C**) and (**D**) SEM images of G-CPE, and (**E**) EDS image of G-CPE.

AFM is a beneficial device for studying the surface morphology of the material at the nano to microscale. The modifier’s average surface roughness (Ra) was determined by AFM analysis [30] and the obtained AFM images are depicted in Figure 1A,B. The Ra of CPE and G-CPE were found to be 0.4 and 1.0 pm^2^, respectively, indicating the enhancement in the surface roughness of the developed electrode material. The SEM of the glucose intercalated carbon matrix (Figure 1C,D) showed an exfoliated and layered structure, which might have been due to the modifier’s uniform dispersion, which led to the increased surface area. The SEM of CPE displayed the homogenous and uniform shape graphite matrix. The SEM image of CPE and SEM-EDS image of CPE are depicted in Figure S1A,B, respectively. Figure S1C displays the EDS image of CPE where we can notice the presence of only one peak for carbon, implying high purity of graphite. The elemental composition of the graphite matrix and modifier was analyzed using energy dispersive X-ray spectroscopy, shown in Figure 1E. The peaks were observed for C and O with the weight percent of 74.46 ± 0.59 (atom%—79.53 ± 0.63) and 25.54 ± 1.28 (atom%—20.47 ± 1.02), respectively.

The outcome of the characterization led to the conclusion that the surface roughness and morphology had improved or increased, due to the incorporation of glucose into the carbon matrix. The EDS image showed the oxygen distribution in the modifier, which later acted as an active site in the sensor mechanism. Based on all of these modifier contributions, it could be concluded that the electrode was effective in detecting trace concentrations of the target analyte.

### 3.2. Electro-Oxidation of TMP

**Figure 2:**
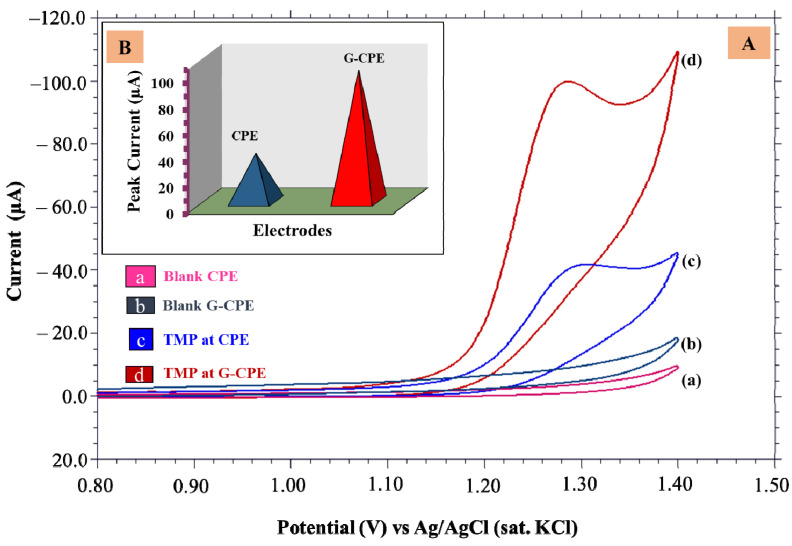
(**A**) Cyclic voltammograms of (**a**) blank CPE, (**b**) blank G-CPE for buffer solution, (**c**) nascent CPE, and (**d**) G-CPE for 0.5 mM TMP solution, (**B**) Peak current responses at fabricated electrodes.

Electrochemical investigations of 0.5 mM TMP at CPE and G-CPE were carried out using the CV technique. A highly defined anodic peak was detected at 1.293 V, having the peak current (Ip) value of 100.3 × 10^−6^ A at G-CPE. While at nascent CPE, an oxidation peak with 36.63 × 10^−6^ A was observed at 1.306 V. During the backward scan, no peak was obtained, indicating the TMP had undergone an irreversible process. It can be noticed from Figure 2 that, in contrast to nascent CPE, G-CPE had higher peak strength.

### 3.3. Accumulation Time

An accumulation time is when the maximum concentration of active species of an analyte molecule interacts with the working electrode. The concentration of the analyte and the contact between the analyte molecule and electrode are key factors in electrochemical processes. The concentration gradient that occurs when the electrode is submerged in the test solution causes the analyte to move toward the electrode before the potential is applied, indicating that the time of the interaction plays a substantial role, influencing the analyte’s vicinity concentration and, consequently, affecting the electrochemical behavior [31]. Hence, to investigate the accumulation time of TMP onto the surface of G-CPE, CV measurements were recorded for 0.5 mM TMP in 3.4 pH of PBS at a definite interval of time from 0 to 30 s at a 0.05 V/s scan rate. It was noticed from Figure S2 that the voltammetric response was highest at the accumulation time of 5 s, which meant that a large concentration of TMP molecules had accumulated at the electrode’s surface, causing the peak current to reach its maximal level. After that, a saturation limit was attained, causing the peak current to decline to show peak current with low values after 5 s. However, the intensified peak current was noticed for 5 s; hence, it was chosen as the optimum accumulation time for the entire investigation.

### 3.4. Impact of Supporting Buffer

**Figure 3:**
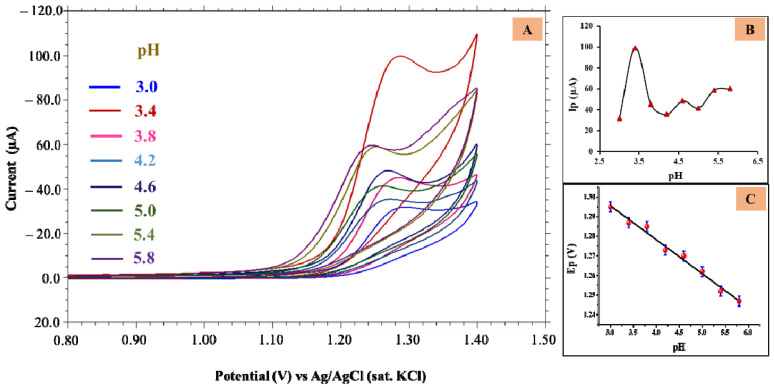
(**A**) Cyclic voltammograms obtained for 0.5 mM TMP at different pH; (**B**) Plot of Ip vs. pH; (**C**) Plot of Ep vs. pH.

The electrochemical behavior of 0.5 mM TMP was examined at various pH levels using cyclic voltammetry in the presence of electrolyte solution with a pH ranging from 3.0 to 5.8 at G-CPE. The recorded voltammograms are depicted in Figure 3A. No oxidation peak was detected for the TMP in and above pH 6.0, suggesting the efficient oxidation of the TMP in the acidic medium for the developed electrode system. The intensified peak was obtained for pH 3.4 (Figure 3B); hence, it was an appropriate buffer for further investigations. From Figure 3C, it is evident that the peak potential (Ep) value was shifting toward a negative potential value with increased pH verifying the transfer of H^+^ ions in the reaction mechanism [32]. The linear equation can be expressed as: Ep = −0.0174 pH + 1.35; R^2^ = 0.987. From the above equation, the slope value was closer to 0.021 V/pH, implying the involvement of unequal protons and electrons in the process [33].

### 3.5. Impact of Scan Rate

The physicochemical characteristics of the electrode process of TMP were examined by increasing the scan rate (υ) from 0.05 to 0.55 V/s using a CV approach for 0.5 mM TMP in pH 3.4. It can be observed from Figure S3A that with the progressive rise in scan rate, a substantial enhancement in the oxidative peak current with shifting peak potential in the direction of positive value evidenced the irreversible nature of the reaction. The Ip and ʋ^1/2^ plot (Figure S3B) shows the linear relationship as follows: Ip (μA) = 197.71 υ^1/2^ (V/s) + 44.15; R^2^ = 0.971.

Diffusion governed the oxidation reaction of TMP, as the obtained slope of 0.395 in the regression equation (log Ip = −0.395 log υ + 2.37; R^2^ = 0.992) for the plot of log Ip versus log υ (Figure S3C) was closely related to the theoretical value of 0.5 [34].

In the case of irreversible reaction, the charge transfer coefficient was evaluated using the Bard-Faulkner Equation (2), and the Laviron Equation (3) was used in estimating the participation of electron number (n) involved in the reaction mechanism [35,36]:

(Ep − Ep/2) = ∆Ep = 47.7/α (mV)(2)

Ep = E° + [2.303 RT/(1 − α)nF]. log[(1 − α)nF/RTk°] + [2.303 RT/(1 − α)nF] log υ(3)

Ep = E° + (0.0591/n) log [(ox)a/(R)b] − (0.0591 m/n) pH(4)

Here, Ep is peak potential, Ep/2 is potential at half of peak current, formal standard redox potential is termed as E°, R is gas constant, T is temperature in K, α termed for charge transfer coefficient, the electron involved in the reaction was termed as n, F is Faraday’s constant, k° is a heterogeneous rate constant, m is number of protons involved and the remnant notations describe their standard meanings. The electron number that participated in the reaction was 1.72 ≈ 2. The proton involved in the reaction was calculated by Equation (4) [36,37], where m is referred to as the proton number. The number of the involved proton (m) was determined to be 0.57 ≈ 1.

The surface coverage concentration (Γ) of TMP at G-CPE was computed using the Equation (5) [38]:

Ip = n^2^F^2^A°Γ_TMP_(υ)/4RT (5)

Here peak current is referred to as Ip, the electrons transferred in the reaction are given by notation n, F is termed for Faraday’s constant, A° refers to the surface area of the working electrode, υ refers to scan rate, R corresponds to gas constant, T refers to the temperature in K, and Γ_TMP_ is the surface coverage concentration of TMP in mol.cm^−2^. ΓTMP at the developed electrode was obtained to be 3.9 × 10^−7^ mol cm^−2^.

### 3.6. Probable Electrode Interaction

**Scheme 1:**
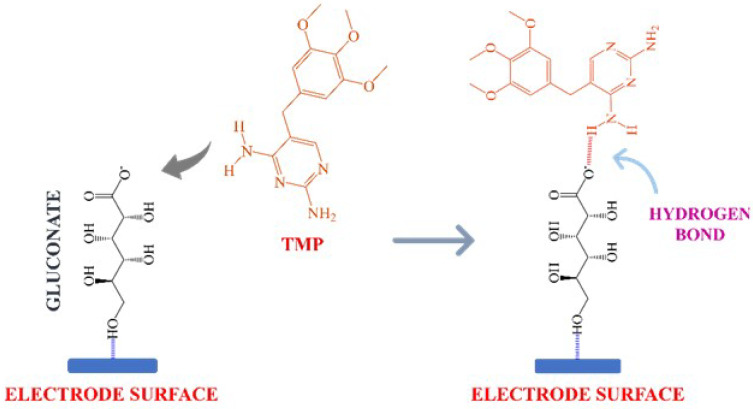
Probable interaction of glucose and trimethoprim.

The working electrode was enriched with a glucose having a hydroxyl group and formed gluconate [26], which conjugated with the analyte through electrostatic force of attraction, which resulted in the formation of the intramolecular hydrogen bond. This interaction was sufficient to hold the TMP in the sensor’s vicinity, as shown in Scheme 1. This temporary coupling of the analyte molecule and the modifier matrix facilitated the electrooxidation reaction mechanism.

### 3.7. Possible Electrode Mechanism

**Scheme 2:**
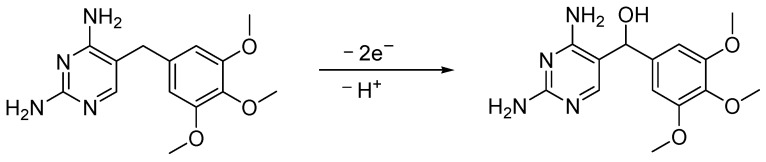
Probable electrooxidation mechanism of TMP at G-CPE.

The result from the investigation of pH suggested the oxidation process involved the participation of protons. From scan rate studies, we acknowledged that an unequal number of protons and electrons were transferred during the reaction. The scan rate study also revealed the electron and proton number, which was determined to be 2 and 1, respectively. The possible electrode mechanism was predicted and is shown in Scheme 2.

## 4. Analytical Applications

### 4.1. Concentration Study

**Figure 4:**
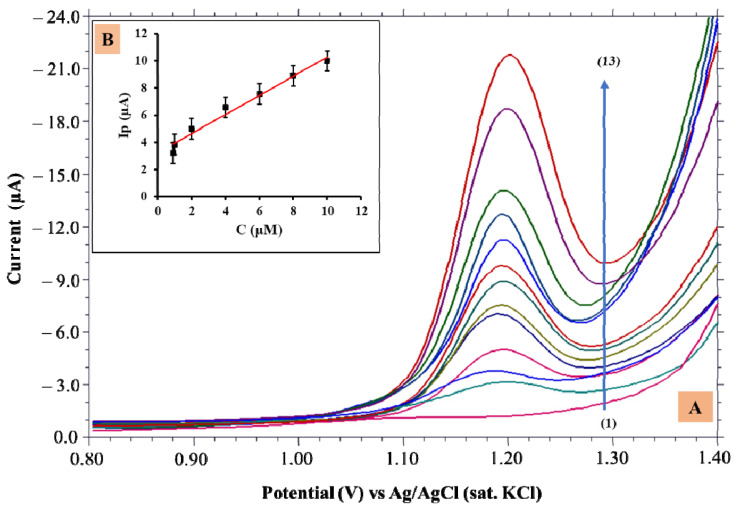
(**A**) Differential pulse voltammograms obtained for various TMP concentrations from 0.9 µM–100 µM (1–13); (**B**) Dependence of Ip vs. concentration.

The CV technique is less sensitive than differential pulse voltammetry (DPV). Detection of TMP in lower concentrations was evaluated employing DPV, and investigations were carried out at pH 3.4 of PBS using the developed electrode. The concentration range varied from 0.9 μM to 100 μM (Figure 4A). The linearity was confined in the concentration range (Figure 4B) viz., 0.9 to 100 μM, respectively. Figure 4B shows that the increase in the TMP concentration enhanced a peak current yielding a linear equation; Ip = 0.709 [TMP] + 3.14; R^2^ = 0.984.

A linear range confined to a lower concentration was selected for quantification. With the help of the standard deviation and slope of the graph, limit of detection (LOD) and limit of quantification (LOQ) were computed by applying the below Equations [39]:

LOD = 3 s/m(6)

LOQ = 10 s/m(7)

The calculated LOD and LOQ were 2.06 × 10^−8^ M and 6.89 × 10^−8^ M, respectively. The calibration characteristics of TMP are shown in Table S1.

The obtained LOD value was compared with the other reported methods (Table 1), which illustrated that the fabricated electrode was more efficient in determining TMP than the other methods.

### 4.2. Effect of Excipients

This research examined the impact of interfering species on TMP determination. Lactose, citric acid, sodium chloride, potassium chloride, and glycine are some excipients in preparing TMP tablets. The excipient solution of appropriate concentration (1 mM) was prepared, and interference of the developed solution was determined for 10 µM TMP using the standard addition method. The obtained reports suggested that the peak current of TMP varied to a certain limit, but it did not exceed the permissible limit of ±5% (Table 2). The excipients did not interfere with TMP peak responses at the G-CPE.

### 4.3. Pharmaceutical Dosage Analysis

The proficiency of the fabricated G-CPE was substantiated by investigating G-CPE with a commercially available TMP tablet utilizing DPV. TMP tablets were finely ground before being dissolved in double distilled water and sonicated. The solution was then subjected to filtration, and the aliquot was diluted to a predetermined amount before being used for recovery studies, employing the standard addition procedure [41,42]. Table 3 displays the recorded analytical data of the TMP tablet solution at G-CPE. Three replicate measurements were recorded for TMP determination for 10 µM of the TMP tablet sample. The recovery obtained verified that the proposed G-CPE had perceptible sensitivity towards TMP. The method illustrated credibility and appropriateness and, thus, could be applied to detect pharmaceutical samples.

### 4.4. Urine Analysis

The proposed sensor was employed to determine the TMP in urine samples by utilizing the DPV technique. For recovery research, a urine sample was collected from healthy individuals and was diluted with water in 1:100 ratios. Then, it was filtered and TMP of a known concentration was added. A known volume of test aliquot in 0.2 PBS of pH 3.4 was taken for the recovery studies. Table 4 shows that the developed electrode had an excellent recovery range toward the TMP and was found to be 95.4–98.78%.

### 4.5. Water Sample Analysis

Water sample analysis was conducted to evaluate the efficiency of the developed sensor to TMP persisting in the environment [43]. The water sample collected from the lake was mixed with an equivalent volume of supporting electrolyte (5.0 mL) and a known amount of TMP was added. The acquired results are shown in Table S2. A technique would need to show sensitivity, selectivity, dependability, minimal cost, ease of handling, and swiftness in providing data to be a valuable instrument for assessing water quality. To build a platform for monitoring environmental water samples, the developed sensor was used to test lake water with the same concentrations of TMP analyzed before. Apart from basic filtering, the water sample was not processed further. The retrieval peak current data suggested that the proposed electrode could be utilized in detecting TMP, even when a complex matrix was present. These findings also supported the G-CPE sensor’s applicability and appropriateness for environmental monitoring.

### 4.6. Stability of G-CPE

To inspect the validity of the developed sensor, repeatability and reproducibility measurements were performed. By taking three consecutive readings with 0.05 mM TMP, the electrode was examined for repeatability. The reports displayed a negligible change in the peak current of TMP, with 95.26% recovery to its initial peak current value. Further, investigations were carried out to check the reproducibility nature of G-CPE. It was placed in a sealed jar for 10 days. Then, the CV scans were acquired, and the reports revealed an excellent recovery response of electrode material with an RSD of 2.74%. Hence, the obtained results verified that the electrode was stable and could give reproducible results.

## 5. Conclusions

The electrooxidation behavior of TMP was investigated by employing a glucose-modified CPE. The current response of TMP was excellent at G-CPE compared to nascent CPE. The AFM and SEM characterization revealed the high surface area and exfoliated layer structure of the modifier, respectively. TMP is electrochemically active in pH 3.0–5.8 of PBS, and the responses were prominent in pH 3.4. The electrode process was irreversible and diffusion-controlled, transferring one proton and two electrons. The concentration linearity was in the range of 9.0 × 10^−7^ to 1.0 × 10^−4^ M. The LOD was found to be 2.06 × 10^−8^ M. Pharmaceutical, spiked urine, and water sample analysis displayed an excellent recovery, which illustrated the suitability of the fabricated sensor in a real-time application. Overall, the sensor was easy to fabricate, cost-effective, and could produce reproducible results. Furthermore, G-CPE allows a miniaturized system to offer high sensitivity and quick response with a small sample volume. Hence, the developed electrode is suitable for drug monitoring in pharmaceutics.

## Figures and Tables

**Table 1:** Comparative study of the limit of detection with some published reports.

Technique	Detection Limit (µM)	Reference
CuPh/PC/GCE	0.6	[5]
HPLC AMP	5.5	[13]
Potentiometric transduction	0.4	[14]
MIP-Gr/GCE	0.1	[15]
CTAB/CPE	0.1	[16]
AgNps-rGo/GCE	0.4	[17]
GR-ZnO/GCE	0.3	[18]
rGNR/SPCE	0.04	[40]
G-CPE	0.02	Proposed method

GCE—glassy carbon electrode, CuPh—copper (II) phthalocyanine/Printex L6 carbon black, HPLC—High-performance liquid chromatography, AMP—Amperometric, MIP-Gr—Molecularly imprinted graphene, CTAB—Cetyltrimethylammonium bromide, CPE—Carbon paste electrode, AgNps—Silver nanoparticles, rGo—reduced graphene oxide, Gr—Graphene, ZnO—Zinc oxide nanoparticles, rGNR/CPE—reduced graphene nanoribbon screen printed carbon electrode, G-CPE—Glucose modified carbon paste electrode.

**Table 2:** Interference studies.

Excipients	Signal Change (I_p_%)
Trimethoprim	0
Lactose	0.06
Citric acid	−1.139
Glycine	−0.229
KCl	1.41
NaCl	1.27

**Table 3:** Tablet analysis.

Trimethoprim	Experimental Findings
Specified amount (mg)	200
Obtained amount (mg) ^a^	187.9
RSD%	0.86
Added (mg) ^a^	1.00
Obtained (mg)	0.93
Recovery%	93.97

^a^ (Average of three measurements).

**Table 4:** Results obtained for analysis of urine samples.

Samples	Spiked (10^−5^ M)	Obtained ^a^ (10^−5^ M)	Recovery (%)
1	1.0	0.95	95.4
2	0.8	0.79	98.7
3	0.6	0.57	96.0

^a^ (Average of three measurements).

## Data Availability

Not applicable.

## Supplementary Materials

The following supporting information can be downloaded at: https://www.mdpi.com/article/10.3390/bios12100909/s1, Figure S1: (A) SEM image of CPE, (B) SEM EDS image of G-CPE, (C) EDS of CPE; Figure S2: Accumulation time; Figure S3: (A) Cyclic voltammograms for varying scan rates from 0.05 mV/s–0.55 mV/s, (B) Plot of peak current vs. square root of scan rate and (C) Plot log (peak current) vs. log (scan rate); Table S1: Calibration characteristics; Table S2: Water sample analysis.
