# Supplementary material for: Glucose Incorporated Graphite Matrix for Electroanalysis of Trimethoprim
Source: Biosensors (Basel). 2022 Oct 21;12(10):909. doi: 10.3390/bios12100909 (PMC9599278; doi:10.3390/bios12100909)
Supplement: Supplementary file 1 [file biosensors-12-00909-s001.zip › biosensors-1943371-supplementary.pdf]

biosensors
MDPI

Supporting Information

Glucose Incorporated Graphite Matrix for Electroanalysis of Trimethoprim

Rakesh R. Sawkar [1], Mahesh M. Shanbhag [2], Suresh M. Tuwar [1,*], Ravindra S. Veerapur [3] and Nagaraj P. Shetti [4,5,*]

[1] Department of Chemistry, Karnatak Science College, Dharwad 580001, India
[2] Department of Chemistry, K.L.E. Institute of Technology, Hubballi 580027, India
[3] Department of Metallurgy & Materials Engineering, Malawi Institute of Technology, Malawi University of Science and Technology, Limbe 5196, Malawi
[4] Department of Chemistry, School of Advanced Sciences, KLE Technological University, Vidyanagar, Hubballi 580031, India
[5] University Center for Research & Development (UCRD), Chandigarh University, Mohali 140413, India
* Correspondence: tuwarsm@kscd.ac.in (S.M.T.); npshetti@kletech.ac.in (N.P.S.)

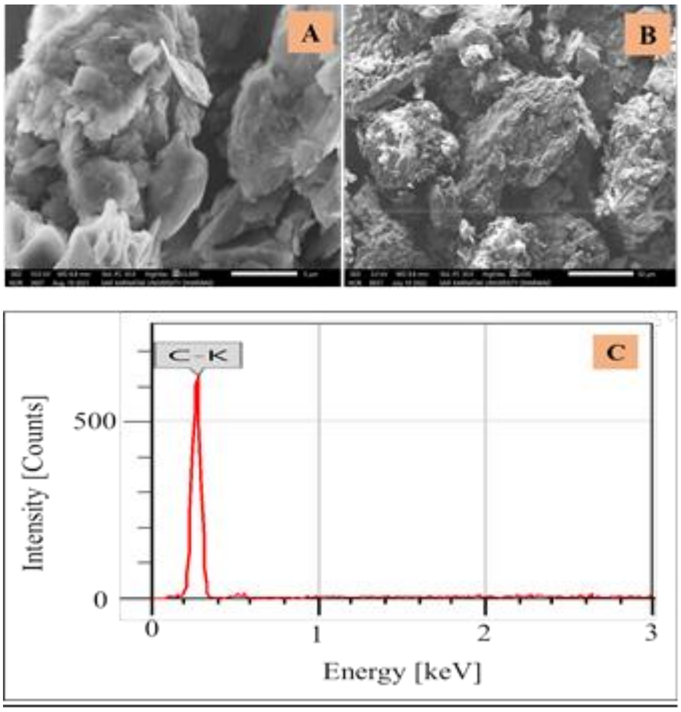

Figure S1. (**A**) SEM image of CPE, (**B**) SEM EDS image of G-CPE, (**C**) EDS of CPE.

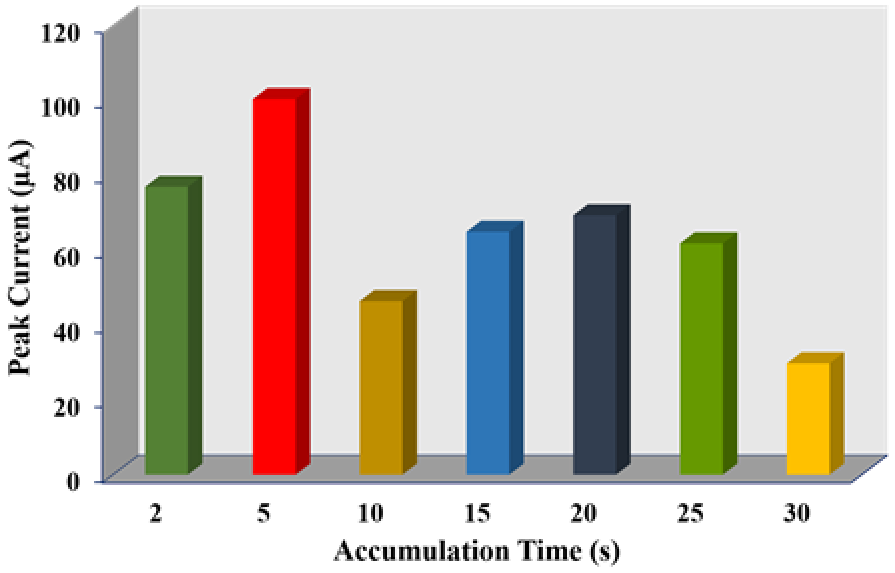

Figure S2. Accumulation time effect.

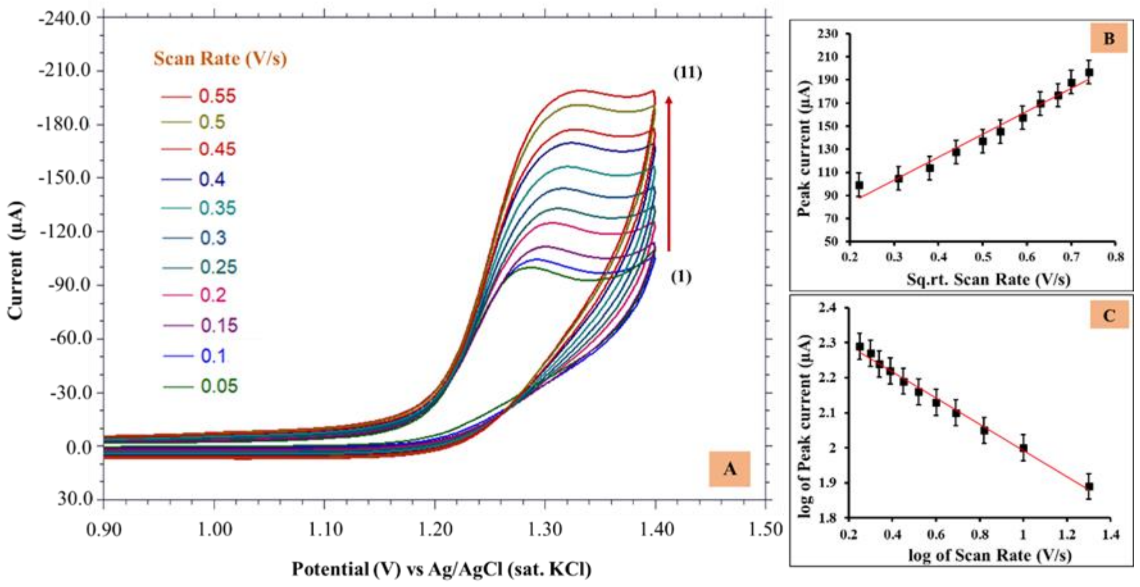

Figure S3. (**A**) Cyclic voltammograms for varying scan rates from 0.05 mV/s – 0.55 mV/s; (**B**) Plot of peak current vs square root of scan rate; (**C**) Plot log (peak current) vs log (scan rate).

Table S1. Calibration characteristics.

Parameter	Observation
Linearity range	0.9 to 100 µM
Slope value	0.709
Intercept value	3.137
Regression co-efficient	0.984
LOD (10^{-8}M)	2.06
LOQ (10^{-8}M)	6.89

Table S2. Water sample analysis.

Water sample	Added (10^{-5} M)	Obtained [a](10^{-5} M)	Recovery (%)
1	0.8	0.78	97.59
2	1.0	0.98	98.08

[a](Average of three determinations).
